# EVALUATION OF TENDON HEALING AFTER ARTHROSCOPIC REPAIR OF ISOLATED SUPRASPINATUS TEARS

**DOI:** 10.1590/1413-785220253302e285693

**Published:** 2025-06-02

**Authors:** Guilherme José Costa Ferreira, Marlos Augusto Bitencourt Costa, Rubens Carneiro dos Santos, Marcos Rassi Fernandes

**Affiliations:** 1 Universidade Federal de Goiás, Faculdade de Medicina, Goias, GO, Brazil.; 2 Hospital Ortopédico de Goiânia, Goiania, Goias, GO, Brazil.; 3 Universidade Federal de Goiás, Faculdade de Medicina, Departamento de Ortopedia e Traumatologia, Goias, GO, Brazil.

**Keywords:** Rotator Cuff Injuries, Arthroscopy, Wound Healing, Magnetic Resonance Spectroscopy, Shoulder, Tendons, Lesões do Manguito Rotador, Artroscopia, Cicatrização, Espectroscopia de Ressonância Magnética, Ombro, Tendões

## Abstract

**Objective:**

To evaluate the healing of supraspinatus tendon lesions after arthroscopic repair, with analysis of intra- and inter-observer agreement by two experienced evaluators for the same lesions.

**Materials and Methods:**

A study was conducted with patients evaluated in the postoperative period of arthroscopic surgery to repair isolated supraspinatus tendon ruptures, with a minimum follow-up of one year. Tendon healing was evaluated using magnetic resonance imaging by two independent evaluators at two time points, with a seven-day interval between them. Categorical data were presented in absolute and relative frequencies, and mixed linear regression models were used to analyze intra- and inter-observer agreement, with a probability of rejecting the null hypothesis of 5%.

**Results:**

Twenty-three patients (26 shoulders) were evaluated, with a mean age of 61.5 years. At both evaluation times, most patients presented a healing grade between I and III in the Sugaya classification for both observers. The interobserver agreement was good, and the intraobserver agreement was excellent.

**Conclusion:**

The mean tendon healing rate for arthroscopic repair of isolated supraspinatus ruptures was 81.7%. Intra-observer agreement ratings were considered excellent, while inter-observer agreement was good, demonstrating reliability in the evaluations performed. ***Level of Evidence II; Cross-sectional study.***

## RESUMO

**Objetivo:**

Avaliar a cicatrização de lesões do tendão supraespinhal após reparo artroscópico, com análise da concordância intra e interobservador por dois avaliadores experientes para as mesmas lesões.

**Materiais e Métodos:**

O estudo foi realizado com pacientes avaliados no pós-operatório de cirurgia artroscópica para reparo das rupturas isoladas do tendão supraespinhal, com seguimento mínimo de um ano. A avaliação da cicatrização tendinosa foi realizada por meio de ressonância magnética, por dois avaliadores independentes, em dois momentos, com intervalo de sete dias entre eles. Dados categóricos foram apresentados na forma de frequências absolutas e relativas e modelos lineares de regressão mista foram utilizados para análise de concordância intra e interobservador, com probabilidade em rejeitar a hipótese nula em 5%.

**Resultados:**

Foram avaliados 23 pacientes (26 ombros), com idade média de 61,5 anos. A maioria apresentou grau de cicatrização entre I e III na classificação de Sugaya para ambos os observadores, nos dois momentos de avaliação. A concordância interobservador foi boa e a intraobservador foi excelente.

**Conclusão:**

A média da cicatrização tendinosa do reparo artroscópico das rupturas isoladas do supraespinhal foi de 81,7%. As concordâncias das avaliações intraobservador foram consideradas excelentes, enquanto a interobservador foi boa, demonstrando confiabilidade nas avaliações. ***Nível de Evidência II; Estudo Transversal.***

## INTRODUCTION

Rupture of rotator cuff (RC) is more common in the elderly due to aging and tissue degeneration and in athletes due to repetitive shoulder movements.^1–3^ Risk factors for these lesions include aging, genetic predisposition, and lifestyle. The disease is multifactorial, with tendon degeneration involving structural changes, characterized by a noticeable change in collagen concentration, especially for a higher prevalence of type III compared to type I.^4^ Muscle atrophy and fat infiltration occur in these chronic lesions and can be observed by imaging techniques, such as magnetic resonance imaging (MRI).^5,6^ There are several surgical techniques for treating tendon ruptures of the RC, each suitable for specific types and severity of lesions, and the choice of the technique is influenced by factors such as the size and retraction of the lesion, tissue quality, and functional needs of the patient.^5,7^ In arthroscopic surgeries, different types of anchors, with various techniques of single or double-row fixation (suture bridge or speed bridge), are recommended to repair broken tendons, and single-row tendons have a higher rate of rupture compared to double-row surgeries, especially in cases of large or extensive ruptures.^7^ The addition of rich plasma in platelets and pluripotentiary cells originating from the bone marrow (speed bridge) during the repair of the RC can favor healing and decrease the index of post-surgical re-ruption.^8^

The healing of the tendon is a complex process, influenced by factors such as age, gender, hyperlipidemia, diabetes mellitus, smoking, fat atrophy/infiltration of the affected muscle, and retraction of the tendon, which can negatively affect this phenomenon.^6,9,10^ The chronicity and size of the injury and the technique used in arthroscopic repair also affect the healing process of the affected tendon and can lead to a re-rupture.^8^

It is observed that there is a divergence in the literature as to the rate of tendon re-rupture of RC, which can reach up to 94.0%.^1,6^ It is also observed that most studies evaluated the healing process of the arthroscopic repair of the ruptures of two or more tendons and not only of the isolated lesion of the supra-spinal, both in single and double rows.^1^ In addition, evaluating the inter and intra-observative agreement of these lesions at different times is of paramount importance for verifying the reliability of the investigated outcome.^11–13^

Thus, the objectives of the present study were to evaluate the healing of arthroscopic repair of isolated ruptures of the supraspinal tendon and verify the agreement between intra- and inter-observative measurements.

## MATERIAL AND METHOD

### Design and location of the study

This cross-sectional study was conducted from June 2022 to February 2024 in a private diagnostic center specializing in orthopedics. The Institute's Research Ethics Committee approved the study under the CAAE number 59403922.2.0000.5078.

### Sampling

The patients were evaluated in the pre-and postoperative video arthroscopic surgery to repair the complete and isolated ruptures of the supra-spinal tendon of the shoulder.

### Eligibility criteria

Patients over the age of 18 were included, with complete and isolated ruptures of the supra-spinal tendon by MRI, operated by arthroscopic technique, by the same surgeon, and with post-operative follow-up of at least one year.

Exclusion criteria were considered: partial ruptures of the RC; the presence of arthrosis or instability in the operated shoulder; involvement of other RC tendons associated with the supra-spinal; performing two or more surgeries to repair the RC in the ipsilateral shoulder; incomplete repair of the rotary sleeve; use of other surgical techniques in the treatment of these lesions, whether muscle transfers, upper capsule reconstruction or reverse shoulder prosthesis.

### Data Collection

A single researcher collected the patients’ sociodemographic and clinical data in a reserved environment after signing the Terms of Free and Informed Consent (TCLE). Subsequently, a Magnetic Resonance Examination of each study participant's operated shoulder was performed. Other preoperative and intraoperative data relevant to the study were listed after verification in medical records.

### Used Classifications

SUGAYA was used to evaluate the structural integrity of the tendon of the supra-spinal muscle of the operated shoulder. It presents five categories in which types I, II, and III refer to the whole sleeve, and types IV and V determine sleeves with re-rupture.^14^

GOUTALLIER was used to evaluate the degree of fat infiltration in the supra-spinal muscle in the preoperative period. It is categorized into different stages that stratify the extension of fat in the muscle in five levels, from the absence of fat (stage G0) to fat predominance (stage G4).^15^

### Image Examination

All operated shoulder MRI tests were performed on the same Siemens brand device, Model Essenza Dot^®^ and Power of 1.5 Tesla. The evaluators were trained on the classification of Sugaya used in the study so that there was a standardization in the analysis of the images in the coronary T2 and sagittal T2 / fat DP sequences. This evaluation was carried out by two professional radiologists with experience in images of the musculoskeletal system, independently and blinded between them, each evaluator being in two distinct moments, with an interval of seven days between them.

### Closures

The study outcome evaluated by the Sugaya classification highlighted above was the tendon healing.

### Independent variables

The following independent variables were assessed: age (in years lived); gender (male / female); side (right / left); smoking (yes / no); diabetes mellitus (yes / no); dominance (right-handed / sinister); postoperative follow-up time (in months); preoperative pseudoparalysis (yes / no); degree of preoperative fatty infiltration (G0/G1/G2 or G3/G4); size of the lesion (small / medium / large); shape of the lesion (crescent / "V" / "U" / "L" / inverted "L" / other); presence of delamination (yes / no); tendon retraction (no retraction / lateral to the glenoid / at the edge of the glenoid / medial to the glenoid); biceps long head tendon (normal / ruptured / degenerated / unstable / degenerated and unstable); biceps tendon procedure (no / tenotomy / tenodesis); type of anchor (non-absorbable / bio-absorbable); number of anchors (in Arabic numerals); type of repair (single lateral row / single medial row / double row); convergence of margins (yes / no); number of stitches (in Arabic numerals); acromioplasty (yes / no); suction cups (yes / no); type of immobilization (simple / abduction); length of immobilization (in weeks).

### Data analysis

The categorical data were presented in the form of absolute and relative frequencies, while the continuous data were presented in the form of averages. The linear mixed regression models were used for intra- and inter-observative concordancy analysis by incorporating the variation between observers and the evaluation moments. From the models, the Intraclass Correlation Coefficients (ICC) were extracted. Random effects models were created, including both the effect of the observers and the evaluation moments for each outcome, plus models for each evaluator, to verify the agreement between their evaluations between the two moments. The following values were adopted as reference for the ICC cutting points: ICC≤0.4 as weak; 0.59≥ICC>0.4 as regular; 0.74≥ICC>0.59 as good and 1.0≥ICC>0.74 as excellent.^16^

The analyses were conducted in the statistical program R, version 4.3.2 (2023, R Core Team, Vienna, Austria). The probability of rejecting the null hypothesis was 5%.

## RESULTS

Of a total of 73 patients with isolated ruptures of the supra-spinal tendon of the shoulder operated by arthroscopy, seven were unavailable for the post-operative MRI examination; 14 were not interested in participating in the study; four had died, and 24 could not be contacted. There was the loss of the MRI images of a patient due to a failure in the data storage system of the diagnostic center, resulting in a final sample of 23 participants and 26 shoulders.

The average age of the participants was 61.0 years (DP: ±7.6). They were all right. There were no patients with pseudoparalytic shoulder. In all procedures, acromioplasty was performed. The average postoperative follow-up time was 50.4 months (DP: ±34.2). The other sociodemographic, clinical/preoperative, and surgical data are shown in Tables 1 and 2.

**Table 1:** Descriptive analysis of the socio-demographic and clinical/pre-operative characteristics of the study participants.

Variables	n	%
**Sex**		
Female	19	82.6
Male	4	17.4
**Side**		
Right	18	69.2
Left	8	30.8
**Smoking**		
Yes	4	17.4
No	19	82.6
**Diabetes**		
Yes	2	8.7
No	21	91.3
**Grade of preoperative fatty infiltration**		
G0/G1/G2	24	92.2
G3/G4	1	3.9
S/D	1	3.9
**Injury size**		
Small	3	11.5
Average	18	69.3
Grande	2	7.7
S/D	3	11.5
**Injury format**		
Rising	15	57.7
U	3	11.5
V	5	19.2
Others	1	3.9
S/D	2	7.7
**Presence of Delamination**		
Yes	1	3.9
No	25	96.2
**Retreat**		
Lateral to Glenoid	9	34.5
On the edge of the Glenoid	1	3.9
No retraction	15	57.7
S/D	1	3.9
**Biceps Long Head Tender**		
Normal	17	65.4
Degenerated	2	7.7
Instable and degenerated	2	7.7
Roto	3	11.5
S/D	2	7.7

S/D – no data. DP – standard deviation.

**Table 2:** Descriptive analysis of the surgical characteristics of the study participants.

Variables	n	%
**Procedure in the tendon CLB**		
Tenodesis	2	7.7
Tenotomy	2	7.7
No	20	76.9
S/D	2	7.7
**Anchor Type**		
Bio-absorbable	11	42.3
Inabsorbable	14	53.8
S/D	1	3.9
**Number of anchors**		
One	17	65.4
Duas	9	34.6
**Repair types**		
Simple lateral filer	5	19.2
Simple medial filer	21	80.8
**Convergence of margins**		
Yes	5	19.2
No	21	80.8
Number of points (average)	3 (DP: ±0.9)	
**Ventures**		
Yes	5	19.2
No	20	76.9
S/D	1	3.9
**Types of immobilization**		
Simples	14	53.8
Abduction	11	42.3
S/D	1	3.9
Immobilization time (average) – in weeks	4.2 (DP: ±0.7)	

S/D – no data. DP – standard deviation.

The Kolmogorov-Smirnov test showed an abnormal distribution for the continuous variables.

Table 3 shows the degree of healing after surgery verified by Sugaya classification of the study participants.

**Table 3:** Evaluation of the degree of healing of the supra-spinal tendon of the shoulder (n=26).

Grade of healing	Evaluator 1		Evaluator 2	
	n	%	N	%
Grade I				
Moment 1	8	30.8	2	7.7
Moment 2	8	30.8	3	11.5
Grade II				
Moment 1	9	34.6	4	15.4
Moment 2	8	30.8	5	19.2
Grade III				
Moment 1	5	19.2	13	50.0
Moment 2	6	23.1	14	53.8
Grade IV				
Moment 1	1	3.8	4	15.4
Moment 2	0	0.0	1	3.8
Grade V				
Moment 1	3	11.5	3	11.5
Moment 2	4	15.4	3	11.5

MRI images of the operated shoulders showed that tendons were healing 84.6% in the first moment and 84.7% in the second moment for Evaluator 1, while for Evaluator 2, it was 73.1% in the first moment and 84.5% in the second moment.

Table 4 demonstrates the intra- and inter-observative agreement regarding the degree of tendon healing of the supraspinal tendon.

**Table 4:** Inter- and intra-observational agreement on the degree of healing of the supra-spinal tendon of the shoulder (n=26).

Model	ICC	p-value	Classification
Inter-observers agreement (moment)			
Evaluator 1	0.895	<0.001*	Excellent
Evaluator 2	0.887	<0.001*	Excellent
Inter-observer concordance (evaluator x moment)	0.740	0.018*	Good

## DISCUSSION

The present study showed that there was healing of the supra-spinal tendon in most of the participants when the arthroscopic repair of the complete and isolated ruptures of the supra-spinal tendon was performed by simple row technique, either medial or lateral, with variation between 73.1% and 84.7%, with the average of the first observer being 84.7% and the average of the second observer being 78.8%. We obtained a healing rate of 81.7% if we consider the averages of the evaluators.

The literature points to a higher rate of tendon re-rupt in postoperative follow-up, which can reach up to 94,0%, depending on the group studied.^1,6,17^ It is therefore highlighted that most previous studies jointly evaluated lesions that did not involve only the supra-spinal tendon of the shoulder, resulting in pre-operative ruptures of two or more tendons and greater potential for re-ruption, compared to those presented in this study.

Fat muscle infiltration before surgical repair and age, especially after age 65, are other important factors in predicting new ruptures.^3,10^ Considering that in this study, we had an average age of 61 years and that most subjects presented low degrees of preoperative fat infiltration, we understand that such risk factors favored the good healing found. Another point to be discussed is the repair technique used, being in the simple queue for all participants. This data differs from the findings of some studies, which showed better healing in repairs, in which the double row or *suture bridge* technique was used when compared to the simple row.^7,18,19^

MRI is recognized as the gold standard in evaluating postoperative healing of RC lesions and provides detailed images of the insertion and tendon structure in addition to the health of the evaluated muscles,^6,11–13^ having high specificity and sensitivity.^20^ The reliability of the operated shoulder MRI images evaluated in our study demonstrated excellent agreement between the evaluations of the same observer at different analysis times and good between them. This inter and intra variability over a short time suggests some difficulty in using the Sugaya classification in evaluating the structural integrity of the tendon lesion repair. It demonstrates the need for continuous improvement by the evaluating professionals to create more ease in the analyses and, therefore, discussion among peers. Other studies that used similar inter and intra-observation comparison methodologies to evaluate tendinosis RC ruptures found similar concordancy rates.^11–13^

As a positive factor of the study, the evaluation of the healing of arthroscopic repair of isolated lesions of the supra-spinal cord is highlighted, associated with the analysis of the agreement of these evaluations made by two experienced professionals for the same lesion at different times. This happened blindly, and the evaluators of the MRI images did not know what had been performed in the intraoperative and independently, without any interference between them.

Despite its retrospective nature, the study collected data in a prospective manner, which increased its level of evidence. In addition, the patients were operated on by the same surgeon and evaluated using the same MRI device, which allowed us to remove the operator's bias and measurement. The surgical technique was similar in all cases, varying depending on the form and size of the injury.

Among the study's limitations, we can cite that, due to the broad exclusive criteria that selected the isolated lesions of the supra-spinal tendon repaired by a specific arthroscopic technique, we had an even reduced sample. The case capture was carried out in a single center, which may indicate a bias in the selection of patients. In addition, the study's design did not allow us to have another comparative surgical group.

## CONCLUSION

The study reveals that healing occurred in most cases, 84.7% on average for Observer 1 and 78.8% on average for Observer 2. If we consider the average of the two observers, the arthroscopic repair tendon healing of isolated supra-spinal rupture was 81.7%. It can also be concluded that the agreements of the intra-professional evaluations were considered excellent and the inter-observation was good, demonstrating reliability in the evaluations performed in the surveyed outcome.
